# Sigma Factor N, Liaison to an *ntrC* and *rpoS* Dependent Regulatory Pathway Controlling Acid Resistance and the LEE in Enterohemorrhagic *Escherichia coli*

**DOI:** 10.1371/journal.pone.0046288

**Published:** 2012-09-27

**Authors:** Avishek Mitra, Pamela A. Fay, Jason K. Morgan, Khoury W. Vendura, Salvatore L. Versaggi, James T. Riordan

**Affiliations:** Department of Cell Biology, Microbiology, and Molecular Biology (CMMB), University of South Florida, Tampa, Florida, United States of America; Centre National de la Recherche Scientifique, Aix-Marseille Université, France

## Abstract

Enterohemorrhagic *Escherichia coli* (EHEC) is dependent on acid resistance for gastric passage and low oral infectious dose, and the locus of enterocyte effacement (LEE) for intestinal colonization. Mutation of *rpoN*, encoding sigma factor N (σ^N^), dramatically alters the growth-phase dependent regulation of both acid resistance and the LEE. This study reports on the determinants of σ^N^-directed acid resistance and LEE expression, and the underlying mechanism attributable to this phenotype. Glutamate-dependent acid resistance (GDAR) in TW14359Δ*rpoN* correlated with increased expression of the *gadX*-*gadW* regulatory circuit during exponential growth, whereas upregulation of arginine-dependent acid resistance (ADAR) genes *adiA* and *adiC* in TW14359Δ*rpoN* did not confer acid resistance by the ADAR mechanism. LEE regulatory (*ler*), structural (*espA* and *cesT*) and effector (*tir*) genes were downregulated in TW14359Δ*rpoN*, and mutation of *rpoS* encoding sigma factor 38 (σ^S^) in TW14359Δ*rpoN* restored acid resistance and LEE genes to WT levels. Stability, but not the absolute level, of σ^S^ was increased in TW14359Δ*rpoN*; however, increased stability was not solely attributable to the GDAR and LEE expression phenotype. Complementation of TW14359Δ*rpoN* with a σ^N^ allele that binds RNA polymerase (RNAP) but not DNA, did not restore WT levels of σ^S^ stability, *gadE*, *ler* or GDAR, indicating a dependence on transcription from a σ^N^ promoter(s) and not RNAP competition for the phenotype. Among a library of σ^N^ enhancer binding protein mutants, only TW14359Δ*ntrC*, inactivated for nitrogen regulatory protein NtrC, phenocopied TW14359Δ*rpoN* for σ^S^ stability, GDAR and *ler* expression. The results of this study suggest that during exponential growth, NtrC-σ^N^ regulate GDAR and LEE expression through downregulation of σ^S^ at the post-translational level; likely by altering σ^S^ stability or activity. The regulatory interplay between NtrC, other EBPs, and σ^N^–σ^S^, represents a mechanism by which EHEC can coordinate GDAR, LEE expression and other cellular functions, with nitrogen availability and physiologic stimuli.

## Introduction

Enterohemorrhagic *Escherichia coli* (EHEC) is an enteric pathogen commonly implicated in food-borne outbreaks of hemorrhagic colitis, and in the life-threatening illness hemolytic uremic syndrome [1]–[3]. To cause disease in humans, EHEC must overcome two formidable innate barriers to infection: the acidity of the stomach, and competition for intestinal colonization sites. For the former, EHEC (and other *E. coli*) has evolved multiple discrete acid resistance mechanisms [4], which allow for survival in highly acidic environments such as the stomach, and which determine a low oral infectious dose [5], [6]. For competitive gut colonization, EHEC utilize a type III secretion system (T3SS) encoded on the locus of enterocyte effacement (LEE) pathogenicity island [7]–[10]. This T3SS translocates EHEC effector proteins into host intestinal cells that mediate intimate attachment to the gut and subvert host cellular processes [11].

The expression of acid resistance and the LEE is influenced by various environmental and intracellular signals, including nutrient availability, stress, and growth phase [12]–[21]. During exponential growth acid resistance is largely repressed, but is activated as cultures transition into stationary phase [13]; for the LEE, the inverse is true [18]. This pattern of expression may reflect the importance of colonization and replication when resources are abundant, and that of stress durability when they are scarce. Many auxiliary regulators communicate these changes in growth conditions to regulatory components of both acid resistance and the LEE [12], [22]–[28]. Alternative sigma factor 38 (σ^S^) is a global regulator that plays an important role in coordinating acid resistance and LEE expression with growth phase. σ^S^ is a protein of low abundance during exponential growth, but accumulates during transition into stationary phase [29]. The acid resistance phenotype of stationary phase cultures is largely attributed to σ^S^ and expectedly, strains mutated for *rpoS* (encoding σ^S^) are sensitive to acid [13], [14], whereas LEE expression is both decreased and increased in response to *rpoS* mutation, depending on growth conditions [28], [30]–[32]. Not surprisingly, *rpoS* mutants are impaired in their ability to survive passage in both murine and bovine models of infection [33]. σ^S^ is tightly regulated at multiple levels of control [34], and the factors that dictate *rpoS*/σ^S^ expression indirectly influence acid resistance, the LEE, and EHEC pathogenesis.

Recently, another alternative sigma factor, sigma N (σ^N^), has been shown to control structural and regulatory genes of both acid resistance and the LEE in EHEC serotype O157:H7 [35]. When bound to RNA polymerase (RNAP), the RNAP-σ^N^ holoenzyme (Eσ^N^) directs transcription from an estimated twenty-one promoters in *E. coli* which specify the transcription of over sixty genes involved in nitrogen and carbon metabolism, and stress resistance [36]–[39]. EHEC strains null for *rpoN* (encoding σ^N^) express elevated levels of acid resistance genes belonging to the glutamate-dependent acid resistance (GDAR) system, and reduced levels of expression for genes encoded on all five operons of the LEE [35]. This altered expression of GDAR and LEE genes is restricted to exponential phase cultures. Furthermore, GDAR upregulation in *rpoN* mutants is correlated with increased survival in acidic environments, and is dependent on an intact *rpoS* gene, suggesting that GDAR is controlled by an as yet uncharacterized σ^N^–σ^S^ regulatory pathway in *E. coli* [35].

There is precedent for such a pathway in *Borrelia burgdorferi*, in which a σ^N^–σ^S^ regulatory pathway controls the expression of membrane lipoproteins essential for transmission and pathogenesis [40]–[42]. In the *B. burgdorferi* model, σ^N^ has been shown to directly activate *rpoS* transcription, which is contrary to *E. coli* in which *rpoS* inactivation abrogates the GDAR phenotype of an *rpoN* null mutant, suggesting that σ^N^ downregulates *rpoS*/σ^S^ by some unknown mechanism. There is evidence that this negative regulation is at the post-transcriptional level, as *rpoN* mutation does not alter *rpoS* mRNA levels [35]. In addition, a recent study reported increased levels and stability of σ^S^ in an *rpoN* mutant of the nonpathogenic *E. coli* strain K-12 MG1655 [43]. This study further explores the regulatory interplay of σ^N^ and σ^S^, and uncovers mechanistic details about σ^N^–σ^S^ directed control of acid resistance and the LEE, and other genetic factors which contribute to the expression of this regulatory pathway.

## Results

### σ^N^–σ^S^ Directed Regulation of Glutamate-dependent Acid Resistance and the Locus of Enterocyte Effacement

Independent regulatory pathways control glutamate-dependent acid resistance (GDAR) genes in response to discrete environmental stimuli through transcriptional modulation of the central regulator *gadE*. These include pathways that stimulate *gadE* during exponential growth in minimal, acidified media (EvgAB-YdeO) [16], [44], or during stationary phase growth in rich media (σ^S^-GadX-GadW) [12], or rich media containing glucose (TrmE) [15]. The growth conditions under which *rpoN*-dependent acid resistance is expressed do not conform precisely to any of these stimulating environments. And yet, mutation of *rpoS* in an *rpoN* null background suppresses GDAR, suggesting that in the WT background σ^N^ negatively regulates GDAR through a σ^S^-dependent pathway; namely, σ^S^-GadX-GadW. To explore this further, transcript levels of GDAR regulatory genes from these activating circuits were measured in WT and mutant backgrounds of TW14359 during exponential growth.

**Figure 1:**
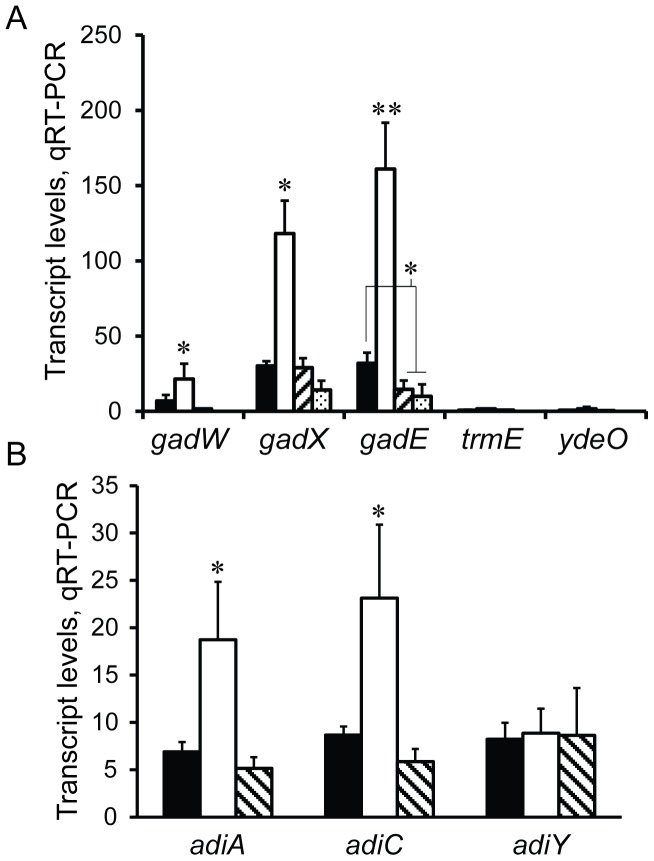
Transcript levels for acid resistance genes. Gene transcript levels as determined by qRT-PCR are plotted for genes of the GDAR system (panel A) and genes of the ADAR system (panel B). Mean transcript levels are normalized to the 16S rRNA gene *rrsH*. Transcript levels are plotted against WT TW14359 (filled), TW14359Δ*rpoN* (empty), TW14359Δ*rpoN* Δ*rpoS* (hatched), and TW14359Δ*rpoS* (stippled, *gadX* and *gadE* only) for panel A. Asterisks denote significant differences by Tukey’s HSD following a significant F-test (n≥3, p<0.05 [*]; p<0.01 [**]). Error bars indicate standard error of the mean.

As anticipated, *gadE* transcript levels were significantly higher in TW14359Δ*rpoN* compared to TW14359 (p = 0.001), as well as TW14359Δ*rpoS* (p = 0.007), and TW14359Δ*rpoN*Δ*rpoS* (p = 0.005) (Fig. 1A). Adding to this, both *gadX* and *gadW* transcripts were upregulated in TW14359Δ*rpoN* (p<0.05), but not in TW14359Δ*rpoS* for *gadX*, or TW14359Δ*rpoN*Δ*rpoS* for either *gadX* or *gadW*. Transcript levels for *trmE* and *ydeO*, key regulators of alternative pathways for *gadE* activation, were in low abundance, and did not differ significantly between strains (Fig. 1A); the presence of amplicons for *trmE* and *ydeO* was validated by gel electrophoresis. Thus, a *rpoN* null mutation leads to increased expression of the GDAR-activating GadX-GadW pathway, agreeing with the *rpoS*-dependency of the phenotype.

In addition to GDAR, σ^S^ regulates at least two more acid resistance systems in *E. coli*: the arginine-dependent acid resistance (ADAR) system [45], and the oxidative-dependent acid resistance (ODAR) system [33]. Both GDAR and ADAR systems protect the cell from acid by a proton scavenging mechanism that is facilitated by the conversion of glutamate to γ-aminobutyric acid (GDAR) or arginine to agmatine (ADAR), and catalyzed by amino acid decarboxylases. ODAR on the other hand does not require glutamate or arginine, and is repressed by glucose [4]. Except for *rpoS*, the regulatory and structural determinants of ODAR are not well understood, and thus were not investigated in this study. For ADAR, the structural genes *adiA* (arginine decarboxylase) and *adiC* (arginine-agmatine exchanger) were slightly but significantly upregulated in TW14359Δ*rpoN* relative to TW14359 and TW14359Δ*rpoN*Δ*rpoS* (p<0.05) (Fig. 1B). However, *adiY*, encoding a putative regulator of *adiA* and *adiC* [46], was not altered in expression in either of the mutant backgrounds. Despite the increase in *adiA* and *adiC* expression in TW14359Δ*rpoN*, there was no corresponding increase in acid resistance by the ADAR mechanism (Table 1), and exclusion of either glutamate or arginine from acidified EG media resulted in no growth for any strains (data not shown). Therefore the only known requirements for *rpoN*-dependent acid resistance are *rpoS*, *gadE*, and glutamate.

**Table 1:** Acid resistance by the GDAR and ADAR mechanisms.

		Percent survival (SD)^a^	
Growth condition	Strain/genotype	GDAR	ADAR
DMEM	TW14359	<0.01^b^	<0.01
	TW14359Δ*rpoN*	24.2 (0.24)	<0.01
	TW14359Δ*fhlA*	21.2 (0.31)	ND^c^
	TW14359Δ*glnG*	15.7 (1.88)	ND
	TW14359Δ*rpoN*Δ*rpoS*	<0.01	<0.01
	TW14359Δ*rpoN* pRAM-1	0.141 (0.11)	0.125 (0.79)
	TW14359Δ*rpoN* pRAM-2	10.61 (1.22)	ND
DMEM +3, 4-DCI^d^	TW14359	<0.01	ND
	TW14359Δ*rpoN*	29.1 (9.3)	ND

a Percent survival by the glutamate-dependent (GDAR) and arginine-dependent (ADAR) acid resistance system; standard deviation (SD).

b Less than 10 CFU/ml remains following 1 h exposure to acidified GDAR or ADAR test environment.

c Not determined (ND).

d DMEM growth media with addition of 5 µM 3,4-dichloroisocoumarin (3,4-DCI).

**Figure 2:**
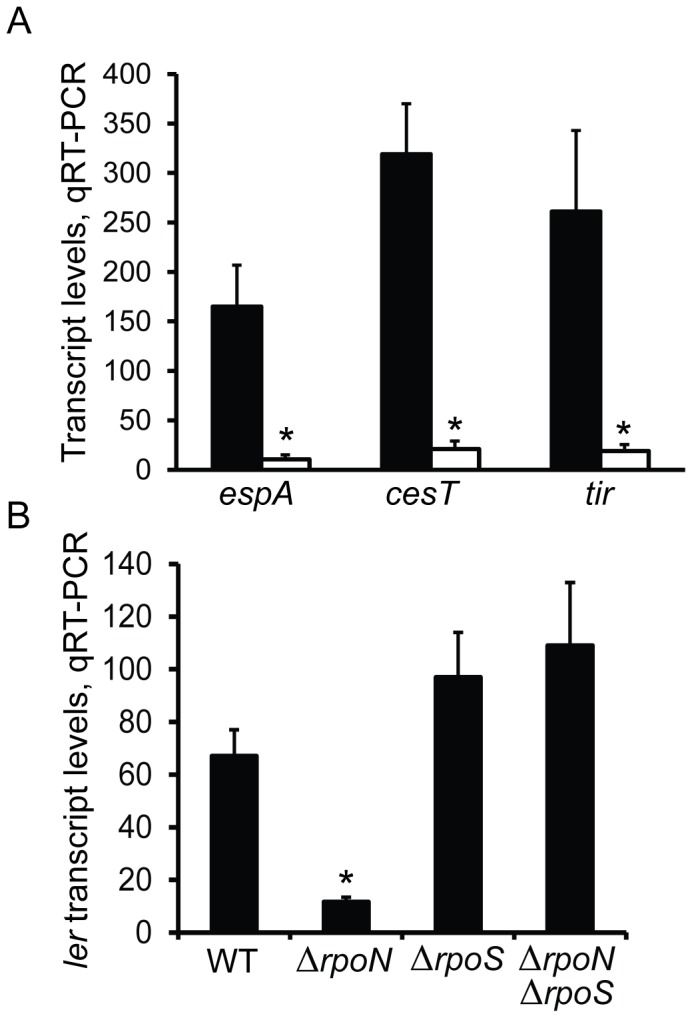
Transcript levels for LEE genes. (Panel A): gene transcript levels as determined by qRT-PCR are plotted for representative LEE genes in WT TW14359 (filled) and TW14359Δ*rpoN* (empty). (Panel B): *ler* transcript levels by qRT-PCR are plotted against TW14359 and various mutant derivative strains of TW14359. Mean transcript levels are normalized to the 16S rRNA gene *rrsH*. For panel A, an asterisk denotes a significant difference between TW14359 and TW14359Δ*rpoN* for each gene by Welch’s t-test (n≥3, p<0.05). For panel B, the asterisk denotes a significant difference between TW14359Δ*rpoN* and the remaining strains by Tukey’s HSD following a significant F-test (n≥3, p<0.05). Error bars indicate standard error of the mean.

σ^S^ has also been shown to upregulate and downregulate transcription of LEE genes in EHEC. For upregulation, σ^S^ is hypothesized to enhance expression of the central regulator of the LEE, *ler* (encoded on operon *LEE1*), in a manner dependent on the non-coding RNA DsrA [28]. It has also been reported that both the *LEE3* and *LEE5* operons possess σ^S^-responsive promoters [30]. For downregulation, σ^S^ is proposed to stimulate an unknown repressor of PchA, which is a positive regulator of *ler* [31], [32], [47]. The mutation of *rpoN* leads to the downregulation of LEE genes during exponential growth [35]. Since σ^N^ controls GDAR through a σ^S^-dependent pathway, it was predicted that σ^N^-directed regulation of the LEE may be similarly dependent on *rpoS*. As expected, transcript levels for LEE genes encoding the T3SS translocon component *espA* (encoded on *LEE4*), the effector chaperone *cesT* (on *LEE5*), and the translocated intimin receptor *tir* (on *LEE5*) were downregulated during exponential growth of TW14359Δ*rpoN* relative to TW14359 (p<0.05) (Fig. 2A). In addition, transcript levels of *ler* (on *LEE1*) were reduced in TW14359Δ*rpoN* compared to TW14359 (p = 0.015) and TW14359Δ*rpoS* (p = 0.011) (Fig. 2B). Importantly, mutation of *rpoS* in TW14359Δ*rpoN* restored *ler* expression to levels consistent with TW14359Δ*rpoS*; *ler* expression was increased in *rpoS* null backgrounds relative to WT, but not significantly increased. These results indicate that σ^N^ positively regulates the LEE during exponential growth in an *rpoS*-dependent manner, and is consistent with the role of σ^S^ as a negative regulator of LEE expression via the PchA-Ler pathway [31], [32], [47].

### Effect of *rpoN* Mutation on *rpoS* mRNA and σ^S^ Stability in EHEC

There is evidence that the mutation of *rpoN* in EHEC does not alter *rpoS* mRNA levels, but instead leads to post-transcriptional alternations in *rpoS*/σ^S^ [35]. The mutation of *rpoN* in *E. coli* strain K-12 MG1655 was recently shown to lead to increased σ^S^ levels and stability [43]. However, there are substantial differences at the genomic level between K-12 and EHEC O157:H7 strains [48]. As an important example, the TW14359 genome (and the genomes of many other EHEC strains), does not contain two of the thirteen σ^N^ enhancer-binding proteins found in K-12 and most other *E. coli*. This study thus aimed to validate the effect of *rpoN* mutation on σ^S^ levels and stability in the EHEC background and under the growth conditions that promote σ^N^-dependent control of GDAR and the LEE.

**Figure 3:**
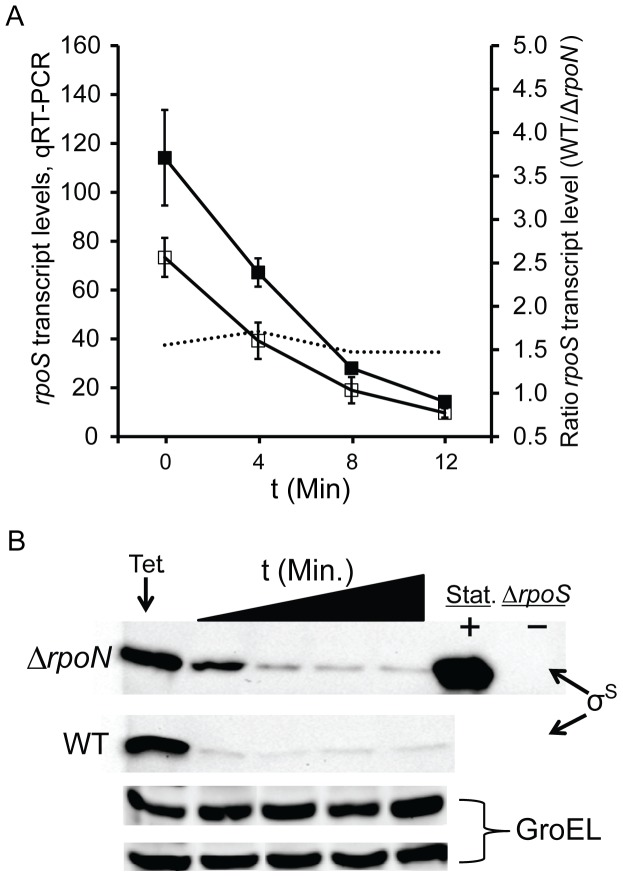
Stability of *rpoS* mRNA and σ^S^. (Panel A): Mean *rpoS* transcript levels (1^st^ ordinate) and ratio of *rpoS* transcript (2^nd^ ordinate) plotted against time following addition of rifampin at t = 0 min for WT TW14359 (filled) and TW14359Δ*rpoN* (empty); ratio is indicated by the dotted line. Error bars denote standard error of the mean (n≥3). (Panel B): Representative western immunoblot for σ^S^ as a function to time following addition of tetracycline at t = 0 min for TW14359 (WT) and TW14359Δ*rpoN* (Δ*rpoN*); blots are in increments of 4 min. Stationary phase (Stat.) protein extracts were used as a positive control for σ^S^, and TW14359Δ*rpoS* (Δ*rpoS*) as a negative control. Equal loading was controlled for by westerns for GroEL (top row is Δ*rpoN*, bottom row is WT).

As anticipated, no difference was observed in the stability of *rpoS* mRNA between TW14359 and TW14359Δ*rpoN* (Fig. 3A). After 12 min of rifampin addition, *rpoS* transcript was barely detectable in both backgrounds and the mean half-life for *rpoS* transcript was estimated at 2.43 min (TW14359) and 2.51 min (TW14359Δ*rpoN*), which agrees with previous estimates [49], [50]. Before addition of rifampin, however, levels of *rpoS* transcript were higher (1.5-fold) in TW14359Δ*rpoN* compared to TW14359, but not significantly higher. In agreement with experiments using strain MG1655, σ^S^ was more stable in TW14359Δ*rpoN* compared to TW14359, however absolute levels were not observed to be higher in TW14359Δ*rpoN* (Fig. 3B) as described for MG1655 [43]. In TW14359, σ^S^ was barely detectable after 4 min of tetracycline addition, but was detected for up to 12 min in TW14359Δ*rpoN*. The mean half-life for σ^S^ was estimated at 2.4 min for TW14359 and 5.5 min for TW14359Δ*rpoN*, increasing by 2.3-fold in the *rpoN* null background. The half-life for σ^S^ has been estimated at 1.4–6.5 min in exponential cultures of *E. coli* [29], [51], [52], and 10.5–30 min in stationary phase cultures [29], [51]. These results reveal that in TW14359Δ*rpoN*, *rpoS*-dependency and control of GDAR and the LEE is correlated with an increase in exponential phase stability, but not absolute levels, of σ^S^.

### Role for Core RNA Polymerase and σ^N^–dependent Transcription in the σ^S^ Stability, GDAR and LEE Expression Phenotype of TW14359Δ*rpoN*

**Figure 4:**
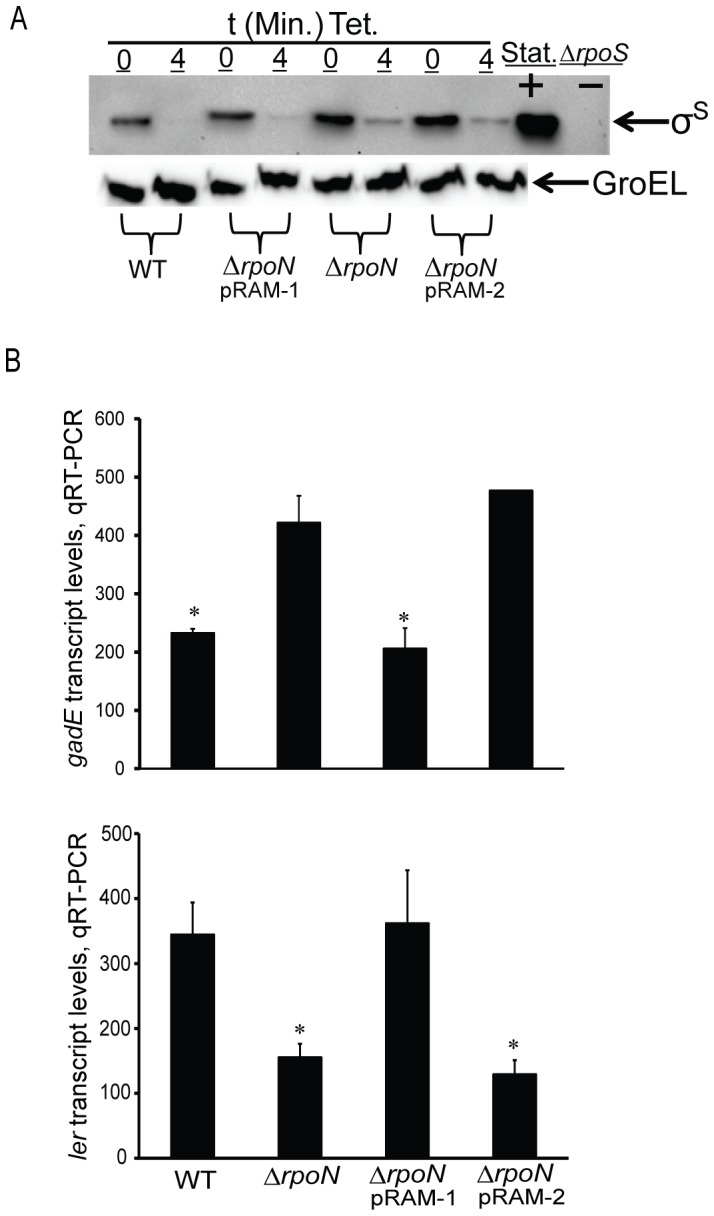
Effect of *rpoN* ^R456A^ expression in TW14359Δ*rpoN* on σ^S^ stability, *gadE* and *ler* transcription. (Panel A): Representative western immunoblots for σ^S^ in TW14359 (WT), TW14359Δ*rpoN* complemented with *rpoN* ^+^ (TW14359Δ*rpoN*pRAM-1), TW14359Δ*rpoN* (Δ*rpoN*), TW14359Δ*rpoN* complemented with *rpoN* ^R456A^ (TW14359Δ*rpoN*pRAM-2) before (t = 0 min) and 4 min after addition of tetracycline (Tet.). Stationary phase (Stat.) protein extracts were used as a positive control for σ^S^, and TW14359Δ*rpoS* (Δ*rpoS*) as a negative control. Equal gel loading was controlled for by westerns for GroEL. (Panel B): Mean *gadE* and *ler* transcript levels by qRT-PCR are plotted against TW14359 (WT) and derivative strains from Panel A. Transcript levels are normalized to the 16S rRNA gene *rrsH*. Asterisks denote significant differences between WT and TW14359Δ*rpoN*pRAM-1 when compared to TW14359Δ*rpoN* and TW14359Δ*rpoN*pRAM-2 by Tukey’s HSD following a significant F-test (n≥3, p<0.05). Error bars indicate standard error of the mean.

The ability of *E. coli* sigma factors to successfully compete for core RNA polymerase (RNAP) differs substantially. For example, the RNAP binding affinity of σ^N^ is second only to the primary sigma factor, σ^70^, whereas σ^S^ binding affinity lies at the bottom of this rank order [53], [54]. In addition, the relative cellular abundance of each sigma factor influences gene expression through competition for RNAP [55]. During exponential growth, σ^N^ levels have been estimated at 10–16% those of σ^70^, whereas σ^S^ is barely detectable [56]–[58]. Together, this suggests that σ^S^ is at a substantial disadvantage for competitive RNAP binding during exponential growth. However, in an *rpoN* null background, the absence of competing σ^N^ may allow for an increase in σ^S^ RNAP binding sufficient enough to protect σ^S^ from ClpXP degradation, leading to increased transcription from σ^S^ promoters. This hypothesis might explain the σ^S^ stability, GDAR and LEE expression phenotype of TW14359Δ*rpoN*. To examine this possibility, a mutant version of the *rpoN* gene (*rpoN* ^R456A^) was constructed, the product of which can efficiently form Eσ^N^ holoenzyme but cannot bind DNA to direct transcription from σ^N^ promoters [91], [92]. If the increased stability of σ^S^ in TW14359Δ*rpoN* is solely the result of increased RNAP binding by σ^S^, the expression of *rpoN* ^R456A^ in TW14359Δ*rpoN* should reproduce WT levels of σ^S^ stability. This was not determined to be the case however, as the stability of σ^S^ in TW14359Δ*rpoN*pRAM-2 did not differ from that of TW14359Δ*rpoN*, and both were increased in comparison to TW14359 and TW14359Δ*rpoN*pRAM-1 (Fig. 4A). The effect of *rpoN* ^R456A^ expression on the GDAR and LEE expression phenotype of TW14359Δ*rpoN* was also examined. Transcript levels for the GDAR regulator *gadE*, and the LEE regulator *ler* in TW14359Δ*rpoN* and TW14359Δ*rpoN*pRAM-2 did not differ, and were significantly higher or lower than TW14359 and TW14359Δ*rpoN*pRAM-1, respectively (p<0.05) (Fig. 4B). Interestingly, survival by GDAR for TW14359Δ*rpoN*pRAM-2 was partially reduced compared to TW14359Δ*rpoN*, but remained substantially higher than TW14359 and TW14359Δ*rpoN*pRAM-1 (Table 1).

### Sensitivity of σ^N^-dependent GDAR and LEE Expression to Protease Inhibition

The low abundance of σ^S^ during exponential growth is due to rapid proteolytic turnover by the serine protease complex ClpXP [29], [51]. In strains mutated for *clpP* (the protease of ClpXP), σ^S^ is completely stable in exponential phase [51], however in exponential phase cultures of TW14359Δ*rpoN*, σ^S^ is still largely unstable (Fig. 3B), suggesting that there remains a sufficient amount of σ^S^ proteolysis. To reproduce the level of increased σ^S^ stability characteristic of TW14359Δ*rpoN* in the WT background, subinhibitory concentrations of the serine protease inhibitor 3, 4-dichloroisocoumarin (3, 4-DCI) [97] were titrated into growing exponential cultures and σ^S^ stability was measured.

**Figure 5:**
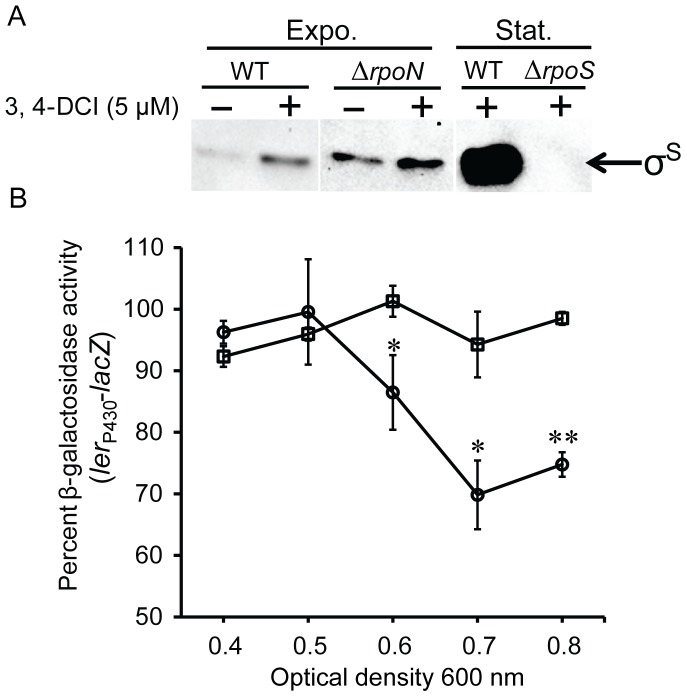
Effect of the serine protease inhibitor 3,4-DCI on σ^S^ stability and *ler* expression. (Panel A): Representative western immunoblot for σ^S^ stability in TW14359 (WT) and TW14359Δ*rpoN* (Δ*rpoN*) during exponential phase (Expo.) 4 min after the addition of tetracycline, and with or without 3,4-DCI, as well as in WT and TW14359Δ*rpoS* (Δ*rpoS*) during stationary phase (Stat.) with 3,4-DCI. Equal gel loading was controlled for by westerns for GroEL. (Panel B): Expression from *ler*_P430_-*lacZ* as measured by mean percent β-galactosidase activity following addition of 3,4-DCI and relative to untreated controls during exponential growth for TW14359 (circles) and TW14359Δ*rpoN* (squares). Asterisks denote significant differences between TW14359 and TW14359Δ*rpoN* at each OD_600_ by Welch’s t-test (n≥3, p<0.05 [*]; p<0.01 [**]).

The addition of 5 µM 3, 4-DCI (or 1/12X MIC) increased σ^S^ stability levels in TW14359 similar to σ^S^ stability levels observed in TW14359Δ*rpoN* without the addition of 3, 4-DCI (Fig. 5A). Addition of 3, 4-DCI further increased σ^S^ levels in TW14359Δ*rpoN* as well, revealing that σ^S^ stability is sensitive to serine protease inhibition in both backgrounds. It was predicted that if the GDAR and LEE expression phenotype of TW14359Δ*rpoN* was simply a result of decreased σ^S^ proteolysis, then experimentally increasing σ^S^ stability with 3,4-DCI should reconstitute a similar phenotype in TW14359. For GDAR this was not shown to be true, as 3, 4-DCI had no impact on survival of TW14359 in acid, and only marginally increased percent survival in TW14359Δ*rpoN* (Table 1). Thus increased stability of σ^S^ alone cannot account for GDAR in TW14359Δ*rpoN*. The expression of LEE genes is known to be positively influenced by ClpP through its proteolytic effect on σ^S^ [31], [32]. Consistent with this, 3, 4-DCI addition reduced expression from *ler*_P430_-*lacZ* in TW14359 as indicated by a decrease in percent β-galactosidase activity relative to untreated controls (Fig. 5B). Since addition of 3,4-DCI further increased σ^S^ stability in TW14359Δ*rpoN*, it was expected that this increase would correspond with a further decrease in *ler* expression. On the contrary, *ler*_P430_-*lacZ* expression did not differ in 3,4-DCI-treated TW14359Δ*rpoN* cultures compared to untreated controls, and β-galactosidase activity was unchanged throughout growth compared to significantly reduced activity in TW14359 (p<0.05) (Fig. 5B). These results reveal that although σ^S^ stability is sensitive to protease inhibition using 3, 4-DCI in TW14359Δ*rpoN*, GDAR and *ler* expression is not and indicates that the underlying mechanism responsible for these phenotypes are at least partially distinct. The addition of 1/2X MIC of 3, 4-DCI did not significantly alter the outcome for GDAR or *ler* expression in either strain (data not shown).

### Identification of the Enhancer-binding Protein Required for σ^N^-directed Regulation of GDAR and the LEE

**Figure 6:**
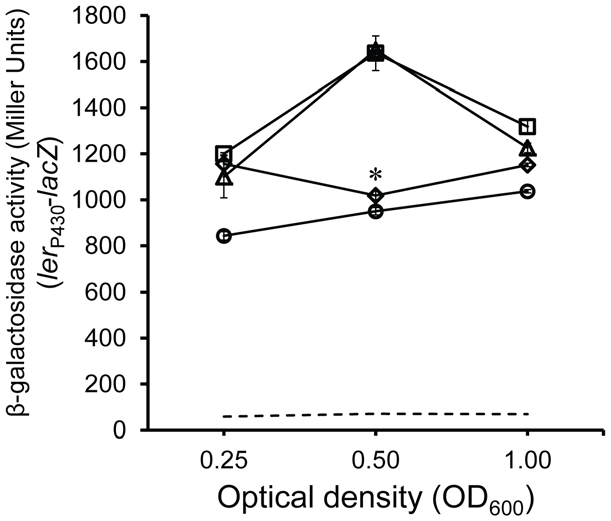
Expression from *ler*_P430_-*lacZ* in σ^N^ enhancer binding protein mutants. Mean expression from *ler*_P430_-*lacZ* represented as β-galactosidase activity during exponential growth for TW14359 (triangles), TW14359Δ*rpoN* (circles), TW14359Δ*fhlA* (squares), TW14359Δ*ntrC* (diamonds) and empty vector pRS551 (hatched line). The asterisk denotes a significant difference for TW14359Δ*rpoN* and TW14359Δ*ntrC* when compared to the remaining strains by Tukey’s HSD following a significant F-test (n≥3, p<0.05).

**Figure 7:**
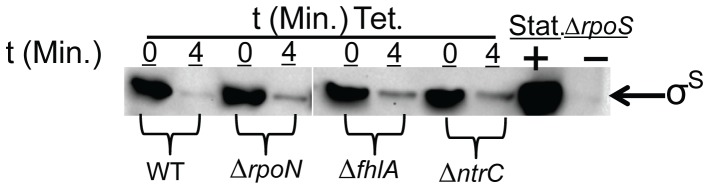
Stability of σ^S^ in σ^N^ enhancer binding protein mutants. Representative western immunoblots for σ^S^ in TW14359 (WT), TW14359Δ*rpoN* (Δ*rpoN*), TW14359Δ*fhlA* (Δ*fhlA*), and TW14359Δ*ntrC* (Δ*ntrC*) before (t = 0 min) and 4 min after addition of tetracycline (Tet.). Stationary phase (Stat.) protein extracts were used as a positive control for σ^S^, and TW14359Δ*rpoS* (Δ*rpoS*) as a negative control. Equal loading was controlled for by westerns for GroEL.

σ^N^ is a unique sigma factor in its requirement for enhancer-binding proteins (EBP) to initiate transcription [59]. If σ^S^ stability, GDAR and LEE expression in TW14359Δ*rpoN* is dependent on σ^N^-directed transcription, at least one of these EBPs is required for this control. To examine this, a library of EBP isogenic deletion mutants in TW14359 was constructed and screened for GDAR during exponential growth. Of the eleven mutants, only TW14359Δ*glnG* and TW14359Δ*fhlA* expressed GDAR comparable to levels observed for TW14359Δ*rpoN* (Table 1). *fhlA* encodes a regulator of formate metabolism [60], and *ntrC* (also *glnG*) encodes NtrC, a major regulator of nitrogen assimilation [61], [62]. The impact of *fhlA* or *ntrC* mutation on LEE expression was then determined by transforming pRJM-1 containing *ler*_P430_-*lacZ* into both EBP isogenic backgrounds, TW14359Δ*rpoN* and TW14359, and β-galactosidase activity was measured during exponential growth. Expression from *ler*_P430_-*lacZ* increased in TW14359 to mid-exponential phase (OD_600_ = 0.5), then tapered off as cells entered late exponential phase (OD_600_ = 1.0) (Fig. 6). For TW14359Δ*rpoN*, *ler*_P430_-*lacZ* expression only slightly increased during growth, and was significantly reduced to 56% of WT levels at OD_600_ = 0.5, concordant with qRT-PCR data (p = 0.008) (Figs. 2 and 6). Mutation of *fhlA* had no apparent effect on *ler*_P430_-*lacZ* expression, yet *ntrC* mutation reduced *ler*_P430_-*lacZ* expression to 50% of WT at OD_600_ = 0.5 (p = 0.006) to levels comparable with TW14359Δ*rpoN* (Fig. 6). Thus the mutation of *ntrC* faithfully reproduces the GDAR and LEE expression phenotype of TW14359Δ*rpoN*. Interestingly, σ^S^ stability was increased in both EBP mutant backgrounds to the level of stability observed in TW14359Δ*rpoN* (Fig. 7). These results reveal that mutation of *fhlA* and *ntrC* similarly influence σ^S^ stability, yet only *ntrC* mutation phenocopies GDAR and LEE expression observed in TW14359Δ*rpoN*. A strain deleted for both *rpoN* and *ntrC* was constructed to validate the dependence on *rpoN* for NtrC-directed GDAR and LEE expression, but the mutant was too growth-impaired in DMEM to be phenotypically informative.

## Discussion

The importance of σ^N^ in *E. coli* metabolism, particularly nitrogen metabolism, is undisputed. Strains mutated for *rpoN* are growth-impaired under nitrogen-limiting conditions due to an inability to activate nitrogen regulatory response promoters. Mutation of *rpoN* also clearly affects many genes in *E. coli* that are not directly tied to metabolism, but which are perhaps cued to the metabolic status of the cell through σ^N^, such as those involved in the regulation of motility [63], [64], NO detoxification [65], and biofilm formation [66]. In the present study, the phenotype of acid resistance and LEE expression previously described for *rpoN* mutants in EHEC [35], represents a case in which σ^N^-dependent regulation is indirectly communicated through the downregulation of another sigma factor, σ^S^. The antagonistic interplay of σ^N^ and σ^S^ in the control of these discrete systems resembles that described on a genomic scale by Dong et al. [43], in which it was estimated that as many as 60% of σ^N^ regulated genes are counter-regulated by σ^S^.

For control of acid resistance, σ^N^ negatively regulates the σ^S^-directed GadX-GadW pathway of glutamate-dependent acid resistance (GDAR) activation. This agrees with the dependence on *rpoS* and *gadE* for acid resistance formerly described for *rpoN* mutants [35], and with research showing that *rpoS* expression in a Δ*gadXW* background cannot induce the GDAR central regulator *gadE* [67]. In this regulatory circuit, σ^S^ drives the transcription of *gadX*, the product of which then activates *gadE* transcription. GadX also downregulates GadW, which is a negative regulator of σ^S^ [12]. As observed for GDAR, σ^N^ is clearly dependent on *rpoS* for upregulation of the LEE, conforming to the role of σ^S^ as a negative regulator of LEE expression [31], [32]. This σ^N^–σ^S^ regulatory pathway is predicted to converge on the LEE central regulator, *ler*. The fact that *ler* expression was not observed to be significantly decreased in previous microarray studies of *rpoN* mutated EHEC [35] but is in the current study, may be explained by the increased sensitivity of qRT-PCR.

The GDAR and LEE expression phenotype of TW14359Δ*rpoN* correlates with an increase in σ^S^ stability similar to that described for K-12 [43], however no increase in σ^S^ levels was observed as was for K-12. This disparity in results could reflect genetic differences between K-12 and TW14359, or differences in experimental growth conditions. For the latter, the M9 glucose media used by Dong et al. [43] should be strongly growth restrictive for *rpoN* mutants, which are auxotrophic for glutamine in minimal media containing glucose [61]. As the production of σ^S^ is sensitive to reduced growth [29], increased σ^S^ levels during growth of *rpoN* mutants in M9 glucose may be attributed to metabolic stress, and not specific to σ^N^. The growth of *rpoN* mutants is impaired in DMEM (Fig. S1), but not prohibitively, as it contains glutamine.

This study further scrutinized the genetic basis for and significance of increased σ^S^ stability in the GDAR and LEE expression phenotype of *rpoN*. The expression of a transcriptionally silent allele of σ^N^ (*rpoN* ^R456A^) in TW14359Δ*rpoN* did not reconstitute WT levels of σ^S^ stability, *gadE* or *ler* expression, suggesting that competition for core RNAP is unlikely to be the primary underlying mechanism for this phenotype, and that transcription from a σ^N^ promoter(s) is a requirement. The RNAP competition hypothesis implies that the simple removal of a competing sigma factor may allow for increased competition of the remaining sigma factors for RNAP core. However, due to the low intrinsic affinity of σ^S^ for RNAP [53], all else being equal, it is more likely that σ^70^, or other sigma factors present during exponential phase (ex. σ^F^) will out-compete σ^S^ for extant core. Naturally, this competition dynamic changes in stationary phase cultures, as small molecules and proteins modulate the ability of specific sigma subunits to interact with RNAP.

Addition of the serine protease inhibitor 3,4-DCI was shown to result in increased σ^S^ stability in TW14359, and further increased σ^S^ stability in TW14359Δ*rpoN*. This cumulative increase in σ^S^ stability in TW14359Δ*rpoN* could reflect the sum of effects of 3,4-DCI and *rpoN* mutation on a common pathway (i.e. ClpP), or independent pathways. There is no direct evidence however, that 3, 4-DCI is increasing σ^S^ stability by inhibiting ClpP. Regardless of which is true, increasing σ^S^ stability alone by interfering with proteolysis did not alter GDAR and LEE expression in TW14359Δ*rpoN*, suggesting that the mechanistic basis of these phenotypes is distinct. Mutation of *rpoN* could lead to increased σ^S^ activity at promoters, or modulate its affinity for RNAP. For the former, both FliZ and 6S RNA have been reported to reduce σ^S^ activity at selective promoters [68], [69]. Interestingly, transcript levels of *fliZ* were markedly upregulated in *rpoN* null K-12 [43], but not in EHEC [35]. For the latter, various proteins and small molecules are known to facilitate Eσ^S^ holoenzyme formation, including Crl [70], Rsd [71], and ppGpp [72]. Currently, the involvement of any of these regulators in σ^N^–σ^S^ control of GDAR and the LEE is unknown.

This study revealed that a strain mutated for *ntrC*, encoding nitrogen regulatory protein NtrC, is phenotypically similar to an *rpoN* mutant in regards to σ^S^ stability, GDAR and LEE expression. NtrC is a canonical σ^N^ EBP, activating transcription from at least 16 promoters in *E. coli* by binding as a hexameric ring to an upstream activator sequence (UAS) distal to the σ^N^−24/−12 binding site [62], [73], [74]. The transcription of *ntrC* dramatically increases when *E. coli* is grown in media that does not contain ammonia (i.e. DMEM), and plays an integral role in controlling nitrogen utilization pathways. This finding suggests that the product(s) of an NtrC/σ^N^ driven promoter directly or indirectly downregulates σ^S^, which in-turn affects GDAR and LEE expression. Currently however, there is no experimental evidence to support a role for any of the known NtrC/σ^N^ regulated genes in this. Alternatively, NtrC could activate σ^N^ promoters independent of DNA binding, which may relax the site selectivity of NtrC/σ^N^ dependent transcription initiation. Examples of this have been described for Rrp2 of *B. burgdorferi*, and FlgR of *Campylobacter jejuni*, that activate σ^N^ promoters in the absence of known UAS sites for these EBPs by some unknown mechanism [75]–[77]. There is also a precedent for NtrC regulating transcription independent of σ^N^. NtrC binds to the core promoters of *glnA*_P1_ and *glnA*_P3_, repressing *glnLG*/*glnALG* (glutamine synthetase operon) transcription by interfering with σ^70^-dependent initiation [61]. Other *E. coli* promoters that are directly downregulated by NtrC have not however been described.

This study further identified FhlA as a putative EBP involved in the control of σ^S^ and GDAR, but not the LEE. FhlA activates transcription from multiple operons involved in formate metabolism, including structural components of the formate hydrogen lysase hydrogenase-3 (Hyd-3) complex. Interestingly, the Hyd-3 complex has been reported to confer acid resistance by a unique mechanism that involves the consumption of protons during the conversion of formic acid to CO_2_ and H_2_ [78]. However, the fact that *fhlA* mutation leads to acid resistance is inconsistent with its role as a positive regulator of the Hyd-3 acid resistance mechanism. Adding to this, Hyd-3 has only been shown to be protective under anaerobic growth conditions [78], together suggesting that the acid resistance conferred by *fhlA* mutation is independent of this mechanism. Alternatively, mutation of *fhlA* may lead to the accumulation of formic acid during growth on glucose (DMEM contains 4 g/l glucose) leading to acid-adaptation. Volatile fatty acid (VFAs, including acetic, formic and butyric acid) production during growth on glucose has been attributed to inorganic acid resistance in *Salmonella* and *E. coli* [79], [80]. The broader significance of this finding is that multiple σ^N^ EBPs regulate GDAR and the LEE by discrete pathways, some of which may be independent of *rpoS*. In further support of this hypothesis, the EBP QseF has been independently shown to be important for attaching and effacing lesion formation, and for the control of T3SS effectors in response to autoinducer 3 (AI-3) and norepinephrine/epinephrine [81]–[83]. The mutation of *qseF* did not however affect GDAR in this study (data not shown).

Given the essential roles of NtrC and σ^N^ in nitrogen metabolism, the results of this study infer that these proteins coordinate the expression of GDAR and the LEE with nitrogen (i.e. NH_3_) availability through σ^S^. This proposed regulatory pathway shares many similarities with that described for *rfaH* expression and O-antigen production in *Salmonella enterica*. Specifically, σ^N^ has been observed to activate *rfaH* transcription in an *rpoS*-dependent manner [84]. However, the mutation of *rpoN* was epistatic for *rfaH* control by σ^S^, indicating a regulatory relationship in which σ^S^ is positively controlling σ^N^; there is no evidence that σ^S^ influences *rpoN*/σ^N^ expression or activity in *E. coli* [35], [43]. Remarkably however, *rfaH* transcription was further determined to be stimulated under nitrogen-limiting conditions [85], which suggests the potential for involvement of NtrC in σ^N^–σ^S^ dependent control of O-antigen production in *S. enterica*.

This study concludes that σ^N^ exerts its regulatory influence on GDAR and the LEE through negative post-translational control of σ^S^. Thus the inactivation of *rpoN* relaxes the requirement for stationary phase-induced mechanisms of σ^S^ accumulation during exponential growth. Furthermore, the results suggest that σ^N^–σ^S^ dependent GDAR and LEE expression is at least partially controlled by NtrC, an EBP that activates transcription from σ^N^ promoters specifying genes for nitrogen utilization. The regulatory interplay of NtrC and other EBPs with σ^N^ and σ^S^ is likely to play a significant role in coordinating transcription with the various nutritional and physiological stimuli EHEC is exposed to during transmission, and in the course of infection.

## Materials and Methods

### Bacterial Strains and Culture Conditions

The strains and plasmids used in this study are listed in Table 2. Strains were stocked at −80°C in glycerol (15% v/v final) diluted in Lysogeny Broth (LB) and were maintained in LB or on LB with 1.5% agar (LBA). Unless otherwise noted, overnight (18–20 h) cultures grown in MOPS (50 mM)-buffered Dulbecco’s Modified Eagle’s Medium (DMEM) (Sigma-Aldrich, cat. #D2902, St. Louis, MO) [86] containing 4 g/l glucose and 4 mM glutamine (pH 7.4) were used to inoculate fresh DMEM to a final OD_600_ = 0.05 and cultured at 37°C on a rotary shaker (200 RPM) using a 1∶10 ratio of media-to-flask volume as described [35]. The growth of strains in DMEM was monitored by taking OD_600_ readings at 1 h intervals over 12 h (Fig. S1). Antibiotics (Sigma-Aldrich) were added to cultures when required. The *rpoS* ^+^ status of strains was confirmed by catalase activity and glycogen storage following previous protocols [87], [88].

**Table 2:** Strains and plasmids used in this study.

Strain/plasmid	Relevant characteristics	Source/reference
*Strain name:*		
DH5α	Vector propagation, *recA1 endA1*	
XL10-Gold®	Competent cells	Agilent, Santa Clara, CA
TW14359	WT 2006 outbreak, western U.S.	[98]
EcRPF-6	TW14359Δ*rpoN*	This study
EcRPF-9	TW14359Δ*rpoN*Δ*rpoS*	This study
EcRPF-7	TW14359Δ*rpoS*	This study
EcRAM-26	TW14359Δ*glnG*	This study
EcRAM-25	TW14359Δ*fhlA*	This study
EcRAM-28	TW14359Δ*qseF*	This study
EcRAM-27	TW14359Δ*pspF*	This study
EcRAM-29	TW14359Δ*ygeV*	This study
EcRAM-4	TW14359*norR*::*kan* Kan^R^	This study
EcRAM-7	TW14359*rtcR*::*kan* Kan^R^	This study
EcRAM-3	TW14359*hyfR*::*kan* Kan^R^	This study
EcRAM-11	TW14359*zraR*::*kan* Kan^R^	This study
EcRAM-8	TW14359*tyrR*::*kan* Kan^R^	This study
EcRAM-5	TW14359*prpR*::*kan* Kan^R^	This study
*Plasmid name:*		
pACYC177	Low copy cloning vector, Amp^R^ Kan^R^ P15A	[99]
pRAM1	*rpoN*::pACYC177, Amp^R^ Kan^S^	This study
pRAM2	*rpoN* ^R456A^::pACYC177 Amp^R^ Kan^S^	This study
pRS551	*lac* fusion vector, Amp^R^ Kan^R^ *lacZ* ^+^ ColE1	[95]
pRJM-1	pRS551 containing *ler*_P430_-*lacZ*fusion	This study

### Directed Gene Deletion and Site-specific Mutation

Gene deletion mutants were constructed using the λ Red recombinase-assisted approach [89], [90] and as described [35]. Primers used for the deletion of σ^N^ EBPs, as well as *rpoN* and *rpoS* are provided in Table S1. For site-specific mutation, a 1,518 bp *Cla*I/*Hind*III-digested PCR fragment containing the *rpoN* gene from strain TW14359 nucleotide positions 4,144,833–4,146,311 was generated using primers rpoN-45/*Cla*I and rpoN+1455/*Hin*dIII (Table S1). This fragment was ligated into *Cla*I/*Hin*dIII-digested pACYC177 to produce pRAM-1 (Table 2). Point mutations C1366G and G1367C were introduced into the *rpoN* gene present on the pRAM-1 template plasmid by PCR using mutagenic primers rpoNR456A-F and rpoNR456A-R (Table S1) and *Pfu Ultra*™ high fidelity DNA polymerase (Agilent, Santa Clara, CA) to produce pRAM-2 (Table 2). The resultant σ^N^ allele has a R456A mutation (*rpoN* ^R456A^) in the DNA binding domain which interferes with the ability of the protein to bind DNA, but does not affect its capacity for RNAP association and holoenzyme formation [91], [92]. pRAM-1, in addition to pRAM-2 purified from *E. coli* XL10-Gold® (Agilent) transformants, were transformed into strain TW14359Δ*rpoN* as described [35]. Genetic constructs were validated by PCR, and restriction mapping, or by DNA sequencing and qRT-PCR.

### Tests for Acid Resistance

Acid resistance by the glutamate- and arginine-dependent systems was measured as described [35] with slight adaptations. For the glutamate-dependent acid resistance mechanism, mid-exponential (OD_600_ = 0.5) DMEM cultures were inoculated to 10^6^ CFU/ml final cell density into E minimal glucose (EG) media with or without 5.7 mM L-glutamate at pH 7 (control) or acidified with HCl (pH 2). To test for arginine-dependent acid resistance, exponential phase DMEM cultures were inoculated into EG media as above but with or without 0.6 mM L-arginine at pH 7 and pH 2.5. EG media acid resistance test environments were incubated at 37°C (200 RPM) for 1 h before sampling. For cell counts (CFU/ml) and percent survival determinations, samples were serially-diluted in PBS (pH 7), plated to LBA and incubated overnight at 37°C.

### Quantitative Real-time PCR (qRT-PCR)

Primers for qRT-PCR are provided in Table S1. RNA purification, cDNA synthesis, qRT-PCR cycling conditions and data analysis followed previously described protocols [35], [93].

### Protein Extraction, SDS-PAGE and Western Immunoblots

To extract total cellular protein, 10 ml culture samples were centrifuged at 10,000×*g* for 2 min and the cell pellet was washed twice with sterile water with centrifugation as above. Washed cell pellets were resuspended in 0.7 ml 0.5 M triethyl ammonium bicarbonate buffer (TEAB) (Sigma-Aldrich) and sonicated with a Sonic Dismembrator 120 (Fisher, Waltham, MA) at 50% amplitude for 30 sec intervals totaling 5 min, followed by incubation at 95°C in 4X Laemmli Buffer for 5 min. Total cell protein was collected from lysed cells by centrifugation at 10,000×*g* for 5 min, and supernatant was removed by aspiration. For western immunoblots, 10–30 µg extracted protein was resolved using 10% SDS-PAGE at 13 V/cm for 80 min before transfer at 15 V for 20 min to polyvinylidene fluoride (PVDF) membranes using a Trans-Blot semi-dry transfer cell (Bio-Rad, Hercules, CA). For detection of σ^S^, PVDF membranes were blocked in Tris-buffered saline (1X Tris, pH 7.4) with 0.1% (v/v) Tween-20 (TBST) containing 5% skim milk for 2 h at room temperature before incubation with anti-σ^S^ mAbs (Neoclone, Madison, WI) diluted 1∶5000 in TBST containing 2% skim milk overnight on a Veri Mix platform rocker (Fisher) at 4°C. Membranes were then incubated for 1 h at room temperature with HRP-conjugated goat anti-mouse pAbs (Bio-Rad) diluted 1∶10,000 in TBST with 2% skim milk. Protein was detected using an enhanced chemiluminescence (ECL) Plus detection system (Amersham-Pharmacia, Piscataway, NJ) following the manufacturer’s instructions. Protein levels were measured and analyzed using a ChemiDoc XRS and Image Lab Software (Bio-Rad). The amount of protein loaded was measured using a Bradford protein assay standard curve. Equal loading was validated by western blots for GroEL using anti-GroEL mAbs (Bio-Rad) diluted 1∶40,000 in TBST with 2% skim milk. Western blots were repeated a minimum of three times in independent trials.

### σ^S^ and *rpoS* mRNA Stability

Cultures were grown to mid-exponential phase (OD_600_ = 0.5) before the addition of a subinhibitory concentration of the transcription inhibitor rifampin (300 µg/ml final) or the translation inhibitor tetracycline (60 µg/ml final). Sampling was performed immediately before addition of antibiotics, and at 4 min intervals thereafter for 12 min (*rpoS* mRNA stability) or 16 min (σ^S^ protein stability). RNA was purified and validated as described [93]. For *rpoS* mRNA stability, gene transcript levels were measured using qRT-PCR and primers rpoS+356 and rpoS+466 (Table S1). Protein was extracted, and σ^S^ levels measured by western immunoblots. The half-life in minutes for *rpoS* mRNA and σ^S^ was extrapolated from gene transcript or protein levels, respectively, using linear regression analysis and as described [94]. The strength of linearity was estimated by the correlation coefficient (r^2^), and exceeded 0.85 (85%) for all analyses.

### *lacZ* Transcriptional Fusions and β-galactosidase Assay

A 429-bp *Bam*HI/*Eco*RI digested PCR fragment generated using primers ler-1/BamHI and ler-430/EcoRI (Table S1) and corresponding to nucleotide positions 4,679,303-4,679,731 in strain TW14359 was cloned into the similarly digested vector pRS551 [95] using T4-DNA ligase (Fisher) to create pRJM-1 (Table 2). This cloned fragment included 429-bp upstream of the translation initiation codon for *ler* (ECSP_4703) and both *ler* P1 and P2 promoters transcriptionally fused to *lacZ* (*ler*_P430_-*lacZ*). pRJM-1 purified from DH5α transformants was used for transformation into various WT and mutant backgrounds. The *ler*_P430_-*lacZ* fusion was confirmed by PCR and sequencing. To measure β-galactosidase activity from *ler*_P430_-*lacZ*, 50 µl culture samples taken at OD_600_ = 0.25 (early exponential), OD_600_ = 0.5 (mid-exponential) and OD_600_ = 1.0 (late exponential) were immediately added to 950 µl Z-buffer (1 M KCl, 1 mM MgSO4, 0.05 M β-mercaptoethanol, 0.06 M Na_2_HPO_4_, 0.04 M NaH_2_PO_4_⋅H_2_O, pH 7) with 0.1 ml chloroform and 50 µl 0.1% (v/v) SDS) and mixed vigorously for 30 sec. Samples were then incubated static at 28°C for 5 min before addition of 0.2 ml ortho-nitrophenyl β-D-galactopyranoside (ONPG, 4 mg/ml in 0.1 M phosphate buffer, pH 7) at 28°C for 20 min. Following development of the yellow cleavage product orthonitrophenol, the reaction was terminated by the addition of 0.5 ml Stop Solution (1 M Na_2_CO_3_) and samples were mixed and then centrifuged at 21,000×*g* for 5 min before measuring β-galactosidase activity. β-galactosidase activity was converted to Miller Units as described [96].

### Serine Protease Inhibition

Selective inhibition of serine protease activity was performed using subinhibitory concentrations (i.e. 1/12X minimum inhibitory concentration (MIC) or 5 µM) of 3, 4-dichloroisocoumarin (3,4-DCI) (Sigma-Aldrich) [97]. The MIC for 3,4-DCI was at 60 µM for both WT and *rpoN* null backgrounds. The effect of 3,4-DCI addition to growing cultures on σ^S^ stability, GDAR and LEE expression was determined as described above. For σ^S^ stability, 3, 4-DCI was added to cultures at mid-exponential phase (OD_600_ = 0.4) and incubated to OD_600_ = 0.5 before addition of 60 µg/ml tetracycline. Sampling was performed immediately before tetracycline addition and 4 min after addition. For GDAR and LEE expression, 3,4-DCI was added at OD_600_ = 0.4 as for σ^S^ stability, and then GDAR tested, or β-galactosidase activity measured from *ler*_P430_-*lacZ* as described above. Control cultures did not contain 3, 4-DCI for all experiments.

## Supporting Information

Figure S1 **Growth of strains in Dulbecco’s Modified Eagle’s Medium (DMEM).** Mean (n = 2) optical density 600 nm (OD_600_) plotted for TW14359 (empty squares), TW14359Δ*rpoN* (filled squares), TW14359Δ*rpoS* (circles), TW14359Δ*fhlA* (plus signs), TW14359Δ*ntrC* (triangles), and TW14359Δ*rpoN*Δ*rpoS* (diamonds). Individual OD_600_ measurements for each strain varied by less than 5%. For *ler*_P430_-*lacZ* expression (Fig. 6), sampling was done for all strains except for TW14359Δ*rpoS* and TW14359Δ*rpoN*Δ*rpoS* at OD_600_ = 0.25, OD_600_ = 0.5, and OD_600_ = 1.0 approximately corresponding to early-, mid- and late-exponential phase, respectively. For all remaining experiments, sampling was done at OD_600_ = 0.5.
(TIF)

Table S1 **Primers used in this study.**
(PDF)
