# Supplementary figures and images for: Sigma Factor N, Liaison to an ntrC and rpoS Dependent Regulatory Pathway Controlling Acid Resistance and the LEE in Enterohemorrhagic Escherichia coli
Source: PLoS One. 2012 Sep 27;7(9):e46288. doi: 10.1371/journal.pone.0046288 (PMC3459932; doi:10.1371/journal.pone.0046288)

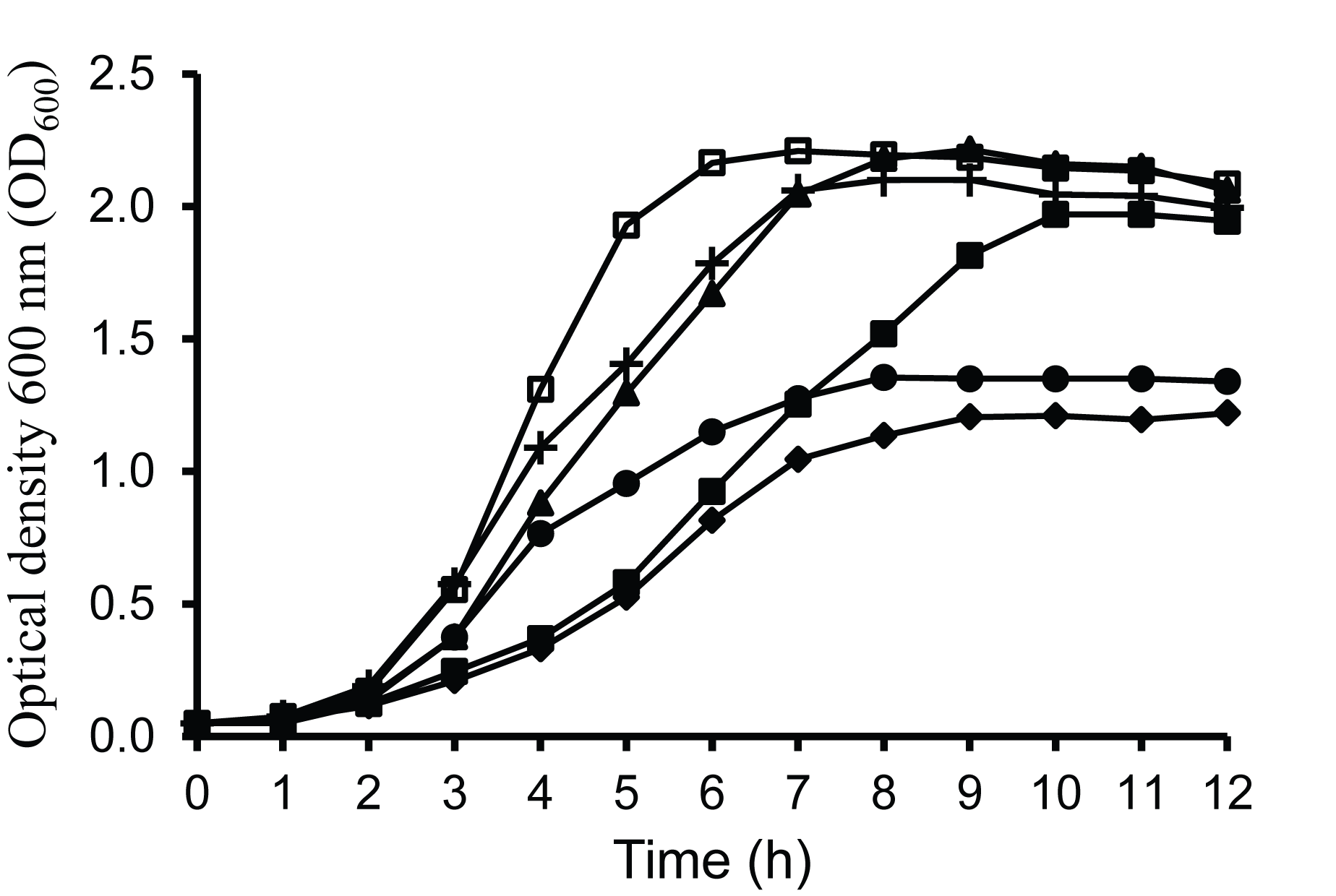

Supplement: Figure S1 — Growth of strains in Dulbecco’s Modified Eagle’s Medium (DMEM). Mean (n = 2) optical density 600 nm (OD600) plotted for TW14359 (empty squares), TW14359ΔrpoN (filled squares), TW14359ΔrpoS (circles), TW14359ΔfhlA (plus signs), TW14359ΔntrC (triangles), and TW14359ΔrpoNΔrpoS (diamonds). Individual OD600 measurements for each strain varied by less than 5%. For ler P430-lacZ expression (Fig. 6), sampling was done for all strains except for TW14359ΔrpoS and TW14359ΔrpoNΔrpoS at OD600 = 0.25, OD600 = 0.5, and OD600 = 1.0 approximately corresponding to early-, mid- and late-exponential phase, respectively. For all remaining experiments, sampling was done at OD600 = 0.5. (TIF) [file pone.0046288.s001.tif]
