# Supplementary material for: Sigma Factor N, Liaison to an ntrC and rpoS Dependent Regulatory Pathway Controlling Acid Resistance and the LEE in Enterohemorrhagic Escherichia coli
Source: PLoS One. 2012 Sep 27;7(9):e46288. doi: 10.1371/journal.pone.0046288 (PMC3459932; doi:10.1371/journal.pone.0046288)
Supplement: Table S1 — Primers used in this study. (PDF) [file pone.0046288.s002.pdf]

Table S1 Primers used in this study.

**Primer name**	**Sequence (5'→ 3')[a]**	**Source**
qRT-PCR:		
gadE+309	TGGTAAACACTTGCCCCATA	[36]
gadE+419	AGCGTCGACGTGATATTGCT	[36]
gadX+319	CGCTATGCAGAAATGCTACG	This study
gadX+413	ACGTTCAGAAGCAGCGGTAT	This study
gadW+445	ATCGCCAAACGTTGGTATCT	This study
gadW+536	CAGGTGTTTTCATCCTGCAA	This study
adiC+987	CGTCGGTATTTTGATGACCA	This study
adiC+1076	ATGACCGACACGGAAGAAAC	This study
adiY+276	CCTGACACCAGACGTCTTTC	This study
adiY+357	GCGTGTTCGTTCCTTTTCTG	This study
adiA+1463	CACAAACCGGCAAAACCTAT	This study
adiA+1542	ATGCATTACCCAGCAGTCCT	This study
trmE+991	ACCGTGGTACGCAATAAAGC	This study
trmE+1083	AGTCTTTGCCGAGAGACGAA	This study
ydeO+548	TAGATGCCAGAATGCAGCAC	This study
ydeO+631	TGGCATAACCACATTGTTCG	This study
rpoS+356	TATCGAAGAGGGCAACCTGG	[36]
rpoS+466	GTTCAATCGTCTGGCGAATC	[36]
ler+109	CGAGAGCAGGAAGTTCAA	[36]
ler+214	GTCCATCATCAGGCACAT	[36]
tir+664	ACTTCCAGCCTTCGTTCAGA	[36]
tir+869	TTCTGGAACGCTTCTTTCGT	[36]
cesT+296	TCCCTCTCGATGATGCTACC	[36]
cesT+445	TGTCGCTTGAACTGATTTCCT	[36]
espA+128	AGGCTGCGATTCTCATGTTT	[36]
espA+310	GAAGTTTGGCTTTCGCATTC	[36]
Cloning:		
ler-430/EcoRI	CGGAATTCGGATTCACTCGCTTGCCGCC	This study
ler-1/BamHI	CGGGATCCAATAAATAATCTCCGCATGC	This study
rpoN-45/ClaI	ATTATCGATGGGTAGAAGTTTGCGACGTT	This study
rpoN+1455/HindIII	AACACAAGCTTGTGTCTTCCTTATCGGTTGG	This study
Mutation:		
rpoNR456-F	GGAACAAGGTATCATGGTGGCACGCGCCACTGTTGCGAAGTACCGAGAGTCT	This study
rpoNR456-R	AGACTCTCGGTACTTCGCAACAGTGGCGCGTGCCACCATGATACCTTGTTCC	This study
fhlA-66/P1	CTAAATCTCCTATAGTTAGTCAATGACCTTTTGCACCGCTgtgtaggctggagctgcttc	This study
fhlA+2106/P2	CAGGCAGATCTGTCCGGCAATTTGCAGTTAAATCAATGCCcatatgaatatcctccttag	This study
glnG-50/P1	CATACCGAGTTCTCGGTTTACCTGCCTATCAGGAAATAAAgtgtaggctggagctgcttc	This study
glnG+1503/P2	CCGGGCAAGATCATACTGAACTTATCGGAACAGTAAAGCGcatatgaatatcctccttag	This study
hyfR-43/P1	TTCTCATTAATAAGGACTGTTGATGGCTATGTCAGACGAGgtgtaggctggagctgcttc	This study
hyfR+2004/P2	TGCAAAAGCAGATTACAACACCTCGCGAACCGAGATCCCCcatatgaatatcctccttag	This study
norR-107/P1	ACCTCAATTTATTCAGCGTGTTCTAAAAAGATGTCTTGCTgtgtaggctggagctgcttc	This study
norR+1510/P2	AGTTGTGATGATTTTGTGCCAGTGCCTGACGAATAGTTTCcatatgaatatcctccttag	This study
prpR-117/P1	TAATCCGCAAATATGCGTTTCAGTTAACGTTTCAGGCAATgtgtaggctggagctgcttc	This study
prpR+1676/P2	CCTATGTAAACATCCCCGATGCGTAAGTTTATCGGTGATCcatatgaatatcctccttag	This study

pspF-41/P1	GCAACATGCCAGGATGAATTAGCTAATTACACTAACAAGTgtgtaggctggagctgcttc	This study
pspF+1029/P2	CACGCCGCATCCGGCAAGTTGTATTGCCCAACTTCGCTAAcatatgaatatcctccttag	This study
rtcR-83/P1	TTATATCTTTACGTCCGTAAACGGAGATTTCCCGCAAAGCgtgtaggctggagctgcttc	This study
rtcR+1564/P2	ACGTCAGACCAAAACGCGCCAGGTATTTGCGTAGCCGATCcatatgaatatcctccttag	This study
tyrR-94/P1	TCTTTGTGTCAATGATTGTTGACAGAAACCTTCCTGCTATgtgtaggctggagctgcttc	This study
tyrR+1523/P2	TGGCTTAAGCCATATTCCCGCAACTTATTGGCAATCGCGGcatatgaatatcctccttag	This study
yfhA+20/P1	ATTTATTATTGGTCGATGACGATCCGGGATTGCTGAAACTgtgtaggctggagctgcttc	This study
yfhA+1333/P2	ATTCCTTGAAATCGTTTGCATCCAGCTCGTGTCGGGAAAGcatatgaatatcctccttag	This study
ygeV-77/P1	GAGTTAATATGATCATGATCTGTGAACCATCAACGTCTTCgtgtaggctggagctgcttc	This study
ygeV+1806/P2	CCTGAATTCAGGCCGGATTCACTGATGTTATGTGTTTAACcatatgaatatcctccttag	This study
zraR-14/P1	TATCGATATTCTGGTGGTGGATGATGACATTAGCCACTGCgtgtaggctggagctgcttc	This study
zraR+1312/P2	TTGCCAACAGCGTTTTGCGCGTGATCCCTAACTGACGGGCcatatgaatatcctccttag	This study
rpoS-56/P1	GGAACCAGGCTTTTGCTTGAATGTTCCGTCAAGGGATCACgtgtaggctggagctgcttc	[36]
rpoS+1104/P2	TTGCCGGGTAGGACGCTGACGTGTCTTATCCAGGCGACAAcatatgaatatcctccttag	[36]
rpoN-12/P1	ACGATTCTGAACATGAAGCAAGGTTTGCAACTCAGGCTTAgtgtaggctggagctgcttc	[36]
rpoN+1430/P2	ACGAGCTGTTTACGCTGGTTTGACGGCGGAATGGATAAAGcatatgaatatcctccttag	[36]

[a] Underlined nucleotides indicate restriction sites (cloning primers) or site-specific mutagenic regions (mutation primers).
